# Phenytoin-Associated Lymphoadenopathy Mimicking a Peripheral T-cell Lymphoma

**DOI:** 10.4084/MJHID.2010.028

**Published:** 2010-09-07

**Authors:** Mark E. Johns, Lynn C. Moscinski, Lubomir Sokol

**Affiliations:** 1 SMDC Medical Center, Duluth, MN, 55805; 2 Department of Pathology and Laboratory Medicine, Moffitt Cancer Center, Tampa, FL, 33612; 3 Department of Malignant Hematology, Moffitt, Cancer Center, Tampa, FL, 33612

## Abstract

We report a case of phenytoin-induced pseudolymphoma in a 28-year-old male with a history of autism and seizure disorder. The patient presented with bilateral cervical lymphadenopathy that was shown to be moderately to markedly FDG-avid on a whole body PET/CT scan. Flow cytometry analysis of peripheral blood and bone marrow mononuclear cells detected identical T cell population with aberrant immunophenotype. Additionally, a TCR beta gene was found to be clonally rearranged in both peripheral blood and bone marrow supporting a clonal origin of atypical T cells. However, no such clonal population of T-cells could be detected in a pathologic specimen obtained from an excisional biopsy of one of the patient’s cervical lymph nodes. After discontinuing the patient’s phenytoin, his lymphadenopathy has nearly completely resolved and circulation clonal T cell population disappeared with 12 months of follow-up.

## Case Report:

**Figure 1.**
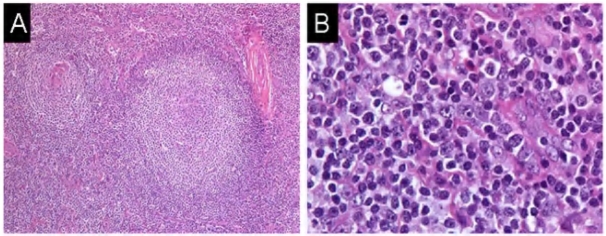

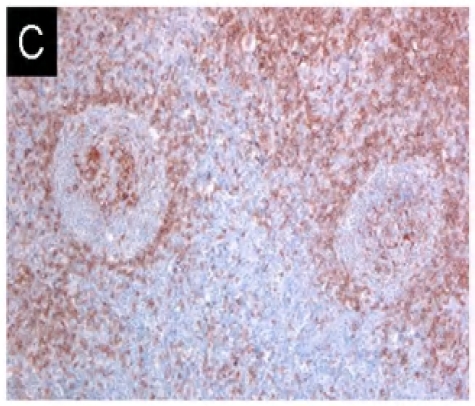
**A.** Follicular atrophy and atypical interfollicular hyperplasia. Partially effaced architecture of lymph node with a nodular proliferation composed of atrophic germinal centers with prominent vasculature accompanied by marked interfollicular expansion. Hypoplastic and sclerotic germinal centers are surrounded and infiltrated by small lymphocytes. (10x) (hematoxylin-eosin) **B.** Atypical interfollicular hyperplasia. Numerous immunoblasts and plasmacytoid lymphocytes are accompanied by small mature lymphocytes and histiocytes. (60x oil immersion) (hematoxylin-eosin). **C.** Immunohistochemistry for CD20. Nodular regions of lymph node consist predominantly of small T-lymphocytes (negative staining) surrounded by a compressed mantle zone and partially intact marginal zone containing CD20+ B-lymphocytes. Hypoplastic germinal centers contain few residual CD20+ lymphocytes. Interfollicular immunoblasts consist predominantly of T-cells. (10x)(CD20 immunoperoxidase)

In June, 2007 a 28-year-old male with a history of autism and seizure disorder treated with phenytoin, was referred to our institution with a diagnosis of angioimmunoblastic T-cell lymphoma. The patient had originally presented to his primary care physician with a complaint of bilateral cervical lymphadenopathy and 4 kg unintentional weight loss in November 2006. After several weeks of observation, an excisional biopsy of a right-sided cervical lymph node was performed. Histology revealed an atypical and partially effaced architecture consisting of a nodular proliferation and a marked interfollicular expansion (**Figures 1**). Flow cytometry and immune-histochemistry did not demonstrate evidence of a B-cell lymphoma. Molecular analysis of the TCR gamma gene failed to reveal a clonal population and molecular analysis of the TCR beta gene was not performed at that time. Nonetheless, based on the histological and immunophenotypic features, a diagnosis of angioimmunoblastic T-cell lymphoma was rendered and the patient was recommended to undergo treatment with combine chemotherapy.

At the time of initial consultation at our institution, the patient’s physical examination was significant for palpable bilateral cervical lymphadenopathy. The largest lymph node was a right-sided level V lymph node that measured 2.9 x 2.0 cm on CT. His abdomen showed no hepatosplenomegaly and his skin was negative for dermatitis. His CBC showed a WBC of 6,400/μL, a platelet count of 324,000/μL, and hemoglobin of 11.8 g/dL. Chemistry panel was unremarkable with normal LDH of 429 U/L.

**Figure 2.**
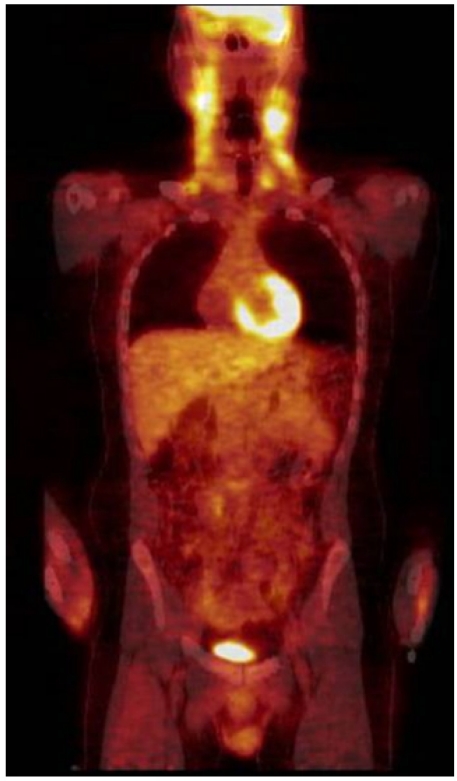
Whole body FDG- PET/CT Scan. Increased FDG-PET uptake in enlarged bilateral cervical lymphonodes.

The whole body PET/CT revealed multiple moderately enlarged lymph nodes throughout the upper, mid, and lower cervical stations bilaterally, and also involving the right tonsillar region. These nodes exhibited moderate to marked hypermetabolic activity (**Figure 2**). No other hypermetabolic foci were noted.

Flow cytometry of the peripheral blood revealed a small T-cell population accounting for approximately 1.5% of the total analyzed cells. These cells lacked expression of CD7 and weakly expressed CD5. Gene rearrangement studies revealed a clonal population with rearrangement of the TCR beta gene. The TCR gamma gene rearrangement study was negative. The bone marrow biopsy and aspirate revealed a normocellular marrow with adequate trilineage maturing hematopoiesis and 14% atypical lymphocytes on a 200 cell differential. Cytogenetics revealed a normal male karyotype, 46, XY. TCR beta gene rearrangement study revealed a clonal population with an identical fragment length to that identified in the peripheral blood. Review of the original pathologic material confirmed the atypical and partially effaced architecture. Clonality studies with TCR gamma and beta genes done on paraffin embedded tissue of lymphonode revealed germinal configuration. Based on the patient’s history of recurrent lymphadenopathy, phenytoin exposure, and inability to demonstrate a clonal population of T cells in the affected lymphonode, a diagnosis of phenytoin-induced pseudolymphoma was rendered and his phenytoin was discontinued. At follow up in June, 2008 his cervical lymph nodes had diminished in size and were approximately 0.5 cm in diameter. Repeat flow cytometry of peripheral blood and TCR gene rearrangement studies showed no evidence of clonal T-cell population.

## Discussion:

Lymphadenopathy in association with the use of hydantoin derivatives such as phenytoin was first described in 1940. Over the years there have been multiple case reports and small series published describing this occurrence and variously terming it Dilantin-associated lymphadenopathy, phenytoin-induced pseudolymphoma, and anticonvulsant hypersensitivity syndrome. More recent publications generally divide these occurrences into two groups: the anticonvulsant hypersensitivity syndrome and phenytoin-induced pseudo-lymphoma. The anticonvulsant hypersensitivity syndrome is a rare syndrome characterized by fever, rash, lymphadenopathy, eosinophilia, and hepatitis. It generally develops within eight weeks after the drug is first prescribed. The presenting symptoms are fever and malaise in most patients. Later, a rash generally starts as a macular erythema involving the upper trunk and face and evolves into a symmetric, pruritic, confluent, papular rash that eventually involves the lower extremities. The lymphoid reaction pattern most commonly seen in this syndrome is a benign lymphoid follicular hyperplasia. Patients generally do well with discontinuation of the anticonvulsant, though corticosteroids have been used in more severe cases. Errore. Il segnalibro non è definito. In contradistinction to the anticonvulsant hypersensitivity syndrome, phenytoin-induced pseudolymphoma is considered to be a late effect of phenytoin therapy, which can occur years after initiating treatment. Errore. Il segnalibro non è definito., Errore. Il segnalibro non è definito. This term applies to those patients with histological and clinical features suggestive of lymphoma. These patients have an atypical lymph node hyperplasia that distorts or effaces the normal lymph node architecture with an atrophic germinal center. Errore. Il segnalibro non è definito. Fever, hepatitis, and eosinophilia are generally absent. Errore. Il segnalibro non è definito., Errore. Il segnalibro non è definito. When a rash is present and a biopsy is obtained, the histological appearance may mimic that of a cutaneous T-cell lymphoma. Errore. Il segnalibro non è definito. Treatment consists of discontinuing Dilantin. Glucocortcoids have been used with success when symptoms persist despite withdrawal of Dilantin. In this case, a PET scan revealed multiple moderately enlarged lymph nodes in the neck that exhibited moderate to marked hypermetabolism. While to our knowledge such hypermetabolism of [18F]-fluoro-2-deoxyglucose (FDG) has not been previously described, this is consistent with another case report which demonstrated increased uptake on a gallium scan. Also particular to this case was the detection of a small monoclonal population of T-cells in the peripheral blood and marrow aspirate, but not in the excised lymph node. The absence of a clonal population of T-cells in the lymph node aided in the diagnosis of phenytoin-induced pseudolymphoma, and it has been suggested that this is the method of choice to distinguish between pseudolymphoma and a T-cell lymphoma. Errore. Il segnalibro non è definito. The detection of a clonal population of T-cells in the peripheral blood of otherwise healthy adults has been reported before and should not be the sole criteria upon which the diagnosis of a T-cell malignancy rests. The mechanisms underlying the development of lymphadenopathy in patients treated with phenytoin are not completely understood. With regards to the anticonvulsant hypersensitivity syndrome, it has been proposed that abnormal detoxification of reactive metabolites of phenytoin, possibly arene oxide metabolites produced by cytochrome P450, is responsible for the syndrome. Errore. Il segnalibro non è definito. Whether the pathophysiology of phenytoin-induced pseudo-lymphoma is similar remains unknown.

In summary, this case report describes the presentation of the rare but important syndrome of phenytoin-induced pseudolymphoma and adds to the available body of literature describing this entity. This case is unique in that the involved lymph nodes were shown to be FDG-avid and that a small clonal population of T-cells was demonstrated in the patient’s peripheral blood and bone marrow, but not in the excised lymph node. Such a case could be easily mistaken for a T-cell lymphoma and should serve as a reminder that careful review of a patient’s clinical and pathologic data are mandatory before initiating cytotoxic chemotherapy.
